# Letter to the Editor Regarding Vaping Article

**DOI:** 10.5041/RMMJ.10380

**Published:** 2019-10-29

**Authors:** Rabbi Yehuda Spitz

**Affiliations:** Yeshiva Ohr Somayach, Jerusalem, Israel

To the Editor,

I am writing in response to Dr Sharon Galper Grossman’s recent fascinating article, “Vape Gods and Judaism—E-cigarettes and Jewish Law.”^1^ The author extrapolates from rabbinic literature regarding combustible cigarettes and suggests that the preliminary data establishing the dangers of e-cigarettes, and the government warnings against usage, would render these products prohibited under Jewish law, especially for youth and pregnant women.

However, as one who has written extensively on the topic of “Smoking and Halacha” and whose article was cited extensively in Galper Grossman’s paper, and although I applaud the author for bringing this important issue to the fore, nonetheless, I believe that such an absolute position is not clear-cut, and, from a *halachic* perspective, several factors may not be as simple as presented.

The author extrapolates from the *Tzitz Eliezer*’s responsum that the leniency of *Shomer Pesa’im Hashem* (G-d protects the simple)^2^ should not apply to e-cigarettes, based on the current Surgeon General’s warning. Yet, and as noted, historically, the *Tzitz Eliezer* himself did not make such an assessment regarding classic cigarettes until far more medical studies were performed and the absolute link from smoking to death was proved. Compare *Shu”t Tzitz Eliezer* from 1945,^3^ to his responsum from 1983,^4^ and finally to his responsum from 1984 where he explicitly prohibited smoking.^5^ Not until 1995 did he address whether those who sell cigarettes and tobacco products are considered to be actively violating *Issurim*.^6^ In other words, although originally of the opinion that it may not be proper to smoke, the *Tzitz Eliezer* held it was still not truly prohibited by *halacha*. Yet, subsequently, and as the knowledge of the health risks associated with smoking became more widespread and universally acknowledged, and the number of smokers starting dropping, only then did he change his *psak* to reflect the emerging reality, using extremely harsh terms to decry smoking, and ultimately outright forbidding it.

Other *Poskim* whose *psak* evolved in accordance with medical findings, gradually becoming more stringent in this matter, include Rav Yosef Shalom Elyashiv,^7^–^10^ Rav Ovadia Yosef,^11^–^13^ Rav Ben Tzion Abba-Shaul,^14^,^15^ Rav Moshe Sternbuch,^16^–^18^ and Rav Menashe Klein,^19^,^20^ all of whose later responsae on the topic were far more restrictive, if not downright prohibiting, than their original *teshuvos* years earlier.

However, and consequently, based on the current reality, with a lacuna in compelling scientific data attesting to the dangers of these products, coupled with the Surgeon General’s rather tepid warning regarding e-cigarettes (merely stating that they contain nicotine and not that they likely cause death, etc., as is currently the case regarding combustible cigarettes), for better or for worse, it appears far from certain that the extrapolation made was indeed accurate.

Although the author opines that *Poskim* “have a unique opportunity to seize this moment to stop e-cigarette use before it becomes widespread,” can one truly expect this? Even the *Tzitz Eliezer*, who was of the most vociferous in his objections to smoking, wrote regarding smoking combustible cigarettes that, as it is deemed enough of a health risk that in every civilized country cigarettes are exclusively sold with a warning printed on the package that it damages health, smoking loses its classification of *Shomer Pesa’im Hashem.* However, regarding e-cigarettes, if the Surgeon General did not see fit to issue a stronger warning for the mainstream public, it would seem quite tenuous to suggest that the *Poskim*—who follow his lead on what is considered hazardous behavior—are obligated to take a more prohibitive stance.

Moreover, as there are different understandings of the *hetter* of *Shomer Pesa’im Hashem*, and since e-cigarettes (at least currently) lack the medical studies to prove that they are just as harmful as classic cigarettes, they may actually still fit this *hetter* at least according to certain opinions (including the *Aruch LaNer*^21^ and Rav Elchonon Wasserman^22^). At this point, even according to all the potential health risks listed in Galper Grossman’s paper, it has not been conclusively proven that vaping is classified as a “*vaday sakana*,” but rather may still fit into the framework of “*chashash sakana*,” which may be then permitted (at least according to several opinions).

Proof of this, perhaps, is that in a recent *Kashrus Magazine* (May, 2019)^23^,^24^ it was reported that Rav Moshe Sternbuch, *Raavad* of the *Badatz Eida Hachareidis*, who is one of the most vocal prohibitors of combustible cigarettes,^17^,^18^ wrote a brief responsa ruling that e-cigarette juice needs *hashgacha* (kashrus certification), as one is receiving tangible benefit from its flavor. Hence, it would seem that, in his opinion at least, there is a quantifiable difference between the levels of *sakana* present in classic versus electronic cigarettes. As he outright, and quite strongly, prohibited classic cigarettes, but not e-cigarettes, writing to make sure to only use those with *hashgacha*, this is implicit proof that he holds they are not in the same *halachic* category.

Regarding the second-hand smoke issue, Rav Moshe Feinstein famously prohibited smoking around others as the smoke was considered *a mazik* (damaging) to them.^25^ However, that ruling was regarding classic cigarettes, where the damage was already proven and people were widely complaining that it bothered them. Currently, as distasteful as vaping may be, the same simply cannot be said about it.

In summation, I commend the author for raising awareness and presenting the dangers and associated health risks of vaping to the public. Obviously, smoking e-cigarettes is an inadvisable and imprudent activity (barring certain specific circumstances detailed in the article), and this certainly holds true from a *halachic* perspective. Yet, in my opinion, it seems—based on the current proven medical knowledge of its intrinsic and inherent risks—it would be somewhat premature at this time to classify it as downright prohibited.
